# Optimization of Biodiesel Production from Waste Cooking Oil Using a Construction Industry Waste Cement as a Heterogeneous and Reusable Catalyst

**DOI:** 10.3390/nano16020108

**Published:** 2026-01-14

**Authors:** Jing Sun, Hongwei Chen, Hongjian Shen, Xiang Luo, Zezhou Lin, Honglei Zhang

**Affiliations:** 1 School of Civil and Transportation Engineering, Ningbo University of Technology, Ningbo 315211, China; sunjingalice@nbut.edu.cn; 2 Ningbo Fenghuan Renewable Energy Co., Ltd., Ningbo 315100, China; chenhw@shenvir.com (H.C.); shenhj@shenvir.com (H.S.); 3 Nottingham Ningbo China Beacons of Excellence Research and Innovation Institute, Department of Chemical and Environmental Engineering, The University of Nottingham Ningbo China, Ningbo 315100, China; xiang-luo@nottingham.edu.cn; 4 Department of Applied Physics, The Hong Kong Polytechnic University, Hong Kong 999077, China; 21037392r@connect.polyu.hk

**Keywords:** biodiesel production, construction industry waste cement, heterogeneous catalyst, transesterification, waste cooking oil

## Abstract

Biodiesel, which is a blend of fatty acid methyl esters (FAME), has garnered significant attention as a promising alternative to petroleum-based diesel fuel. Nevertheless, the commercial production of biodiesel faces challenges due to the high costs associated with feedstock and the non-recyclable homogeneous catalyst system. To address these issues, a solid catalyst derived from construction industry waste cement was synthesized and utilized for biodiesel production from waste cooking oil (WCO). The catalyst’s surface and physical characteristics were analyzed through various techniques, including Scanning Electron Microscopy-Energy Dispersive Spectroscopy (SEM-EDS), X-ray diffraction (XRD), X-ray photoelectron spectroscopy (XPS), and Fourier Transform Infrared Spectroscopy (FTIR). The waste-cement catalyst demonstrated remarkable catalytic performance and reusability in the transesterification of WCO with methanol for biodiesel synthesis. A maximum biodiesel yield of 98.1% was obtained under the optimal reaction conditions of reaction temperature 65 °C; methanol/WCO molar ratio 16:1; calcined cement dosage 3 g; and reaction time 8 h. The apparent activation energy (Ea) from the reaction kinetic study is 35.78 KJ·mol^−1^, suggesting that the transesterification reaction is governed by kinetic control rather than diffusion. The biodiesel produced exhibited high-quality properties and can be utilized in existing diesel engines without any modifications. This research presents a scalable, environmentally benign pathway for WCO transesterification, thereby contributing significantly to the economic viability and long-term sustainability of the global biodiesel industry.

## 1. Introduction

The adverse environmental and economic consequences linked to fossil fuel usage, such as global warming, air pollution, and resource depletion, have driven the need for cleaner, renewable alternatives [1]. Notably, biodiesel has surfaced as one of the most promising substitutes for traditional diesel fuel [2,3]. Biodiesel is mainly produced from vegetable oils, animal fats, or other lipid-rich feedstocks through a chemical process called transesterification, in which triglycerides (fatty acid esters) react with alcohol, usually methanol, to yield biodiesel and glycerol [4,5]. Biodiesel presents numerous benefits, including biodegradability, non-toxicity, and reduced emissions of harmful pollutants when combusted compared to conventional diesel [6,7]. Furthermore, biodiesel can be manufactured locally, which diminishes the reliance on imported fossil fuels and enhances energy security. Consequently, biodiesel production has garnered significant interest from both academic researchers and industrial stakeholders [8].

However, the progress of the biodiesel industry currently faces two major challenges. First, although many companies worldwide have achieved industrial-scale biodiesel production, they often encounter limitations such as limited production capacity, high energy consumption, decreased catalyst activity, and poor biodiesel quality [9]. Second, the production costs of biodiesel are extremely high, with raw material costs accounting for approximately 75% [10,11]. As a result, the use of waste cooking oils as feedstock for biodiesel production has attracted significant attention. Waste cooking oil (WCO), often discarded by households, restaurants, and the food processing sector, is a cost-effective and abundant resource that also helps mitigate environmental problems associated with improper disposal [12,13]. By utilizing WCO for biodiesel production, the environmental consequences of waste cooking oil disposal are diminished, and the production costs of biodiesel are greatly reduced, providing an environmentally sustainable solution to waste management [6]. Nonetheless, the successful conversion of WCO into biodiesel requires a capable and efficient catalyst, which remains a considerable challenge.

Catalysis plays a crucial role in the transesterification reaction, wherein triglycerides react with methanol or ethanol to yield methyl or ethyl esters alongside glycerol. The catalyst accelerates this chemical reaction by lowering the activation energy and enhancing the reaction rate. In biodiesel production, two main categories of catalysts are employed: homogeneous and heterogeneous catalysts [5,14,15]. Homogeneous catalysts, such as sodium hydroxide (NaOH) and potassium hydroxide (KOH), are frequently utilized in transesterification processes due to their high reactivity [16]. However, these catalysts have several drawbacks, including difficulty in recovering and separating from the reaction mixture, which requires extensive purification processes and higher post-treatment costs [17]. Furthermore, the use of homogeneous catalysts can result in environmental pollution and waste-disposal challenges, as they are highly caustic and often produce hazardous by-products. On the other hand, heterogeneous catalysts, which exist in a different phase from the reactants, offer several advantages over their homogeneous counterparts. Heterogeneous catalysts can be easily separated from the reaction mixture, reducing the need for complex post-reaction processing and enabling reuse [18]. Additionally, heterogeneous catalysts are generally more stable under reaction conditions, exhibit fewer side reactions, and can be utilized in continuous-flow processes, making them more suitable for large-scale biodiesel production.

The increasing demand for environmentally friendly and cost-effective catalysts has led to the exploration of alternative materials that can serve as efficient heterogeneous catalysts [19]. One notably promising candidate is waste cement from the construction industry, which originates from by-products of construction processes, such as limestone and other calcium-rich materials [5,20,21]. Primarily composed of calcium oxide (CaO), cement has shown considerable promise as a low-cost, abundant, and eco-friendly catalyst [21]. This catalyst can be modified or calcined to enhance its catalytic efficiency, making it appropriate for transesterification reactions. In recent years, numerous studies have focused on using waste materials as catalysts for biodiesel production, aiming to reduce both the economic and environmental impacts associated with conventional catalysts. Although the use of construction waste cement in biodiesel production is still relatively uncharted, its potential as a sustainable catalyst for the conversion of waste cooking oil is of significant interest. The advantages of utilizing construction industry waste cement as a catalyst include its availability, low cost, and the added benefit of reducing industrial waste, thus promoting a circular economy [21,22,23]. Additionally, this catalyst can be easily regenerated and reused across multiple cycles, improving the economic feasibility of the process. However, these most reported waste-derived CaO systems always require higher catalyst (>5 wt%), higher methanol oil molar ratios (>20), or longer durations [5,20,21].

Moreover, understanding the kinetic behavior of the biodiesel production process is crucial for optimizing reaction conditions and maximizing biodiesel yield. The transesterification process involves various factors, such as temperature, reaction time, catalyst amount, and methanol-to-oil ratio, all of which influence the reaction rate and the final biodiesel yield. To ensure efficient and high-yield biodiesel production, it is imperative to thoroughly investigate the impact of these parameters and determine optimal reaction conditions. Additionally, a thorough kinetic study provides insights into the reaction mechanism, allowing for better process design and scale-up for industrial applications. The activation energy and reaction rate constants are key parameters that can guide the optimization of the process for large-scale biodiesel production.

In this study, a novel heterogeneous catalyst derived from construction industry waste cement is evaluated for its effectiveness in the transesterification of waste cooking oil into biodiesel. The primary objectives of the research are to (1) synthesize a promising solid base catalyst with waste cement from the construction industry; (2) optimize the key parameters involved in the biodiesel production process, including the methanol-to-oil ratio, catalyst dosage, reaction temperature, and duration, using waste cement as a catalyst; (3) conduct a kinetic study to understand the reaction mechanism and determine the activation energy and rate constants; (4) investigate the reusability of the catalyst over multiple cycles; and (5) study the physicochemical properties of the produced biodiesel and compared against the ASTM D6751 [24] and EN 14214 [25] standard. By achieving these objectives, the research aims to develop a cost-effective, environmentally friendly, and renewable biodiesel production process that simultaneously utilizes two waste materials, thereby supporting both waste reduction and renewable energy generation.

## 2. Materials and Methods

### 2.1. Materials

The waste cement (CaCO_3_) was sourced from industrial construction sites, serving as a by-product of cement production. This material underwent three washes to eliminate dirt and other contaminants, followed by drying at 110 °C for 24 h. Methanol with a purity of 99.9% was acquired from Sigma-Aldrich (St. Louis, MO, USA) and utilized as the alcohol reactant in the transesterification process. Waste cooking oil (WCO) was gathered from local restaurants and food vendors. The WCO was subjected to filtration, degumming, and separation to extract any solid particles, soluble salts, and moisture prior to its application in the transesterification reaction. The physicochemical characteristics of WCO, such as free fatty acid (FFA) content, were assessed using standard analytical techniques, and the results are displayed in Table 1.

The composition of the WCO was examined through gas chromatography-mass spectrometry (GC-MS, 6890N, Agilent, Santa Clara, CA, USA); the results are illustrated in Table 2. The unrefined WCO predominantly contains lauric, myristic, palmitic, oleic, and linoleic acids.

### 2.2. Catalyst Preparation

Initially, construction waste cement was ground to a particle size of less than 100 μm to enhance its specific surface area and ensure uniformity. Subsequently, it underwent ultrasonic washing three times with deionized water to eliminate soluble salts and organic substances. Following this, the material was dried at 100 °C for 12 h until a constant weight was reached. The processed material was then placed in a tube furnace and subjected to calcination in air to promote the decomposition of carbonates. The heating rate was maintained between 5 and 10 °C min^−1^, with a calcination temperature set at 800 °C and a holding duration of 2 h. The resulting calcined cement was further sieved to isolate particles within the 100–200 micrometer range. The prepared catalyst was stored in airtight containers to avert moisture absorption prior to its application in the transesterification reaction.

### 2.3. Catalyst Characterization

Scanning electron microscopy (SEM, Gemini SEM 360, Thermo Fisher Scientific, Waltham, MA, USA) was utilized to investigate the morphology of the catalysts. Backscattered electron imaging, along with Energy Dispersive Spectroscopy (EDS), was employed to assess compositional contrast and elemental distribution. The X-ray diffraction (XRD, Bruker D8 Advance) utilizing Cu-Kα radiation (λ = 1.5418 Å) was conducted to analyze the crystal phase at a scanning speed of 2° per minute. X-ray photoelectron spectroscopy (XPS, AXIS Supra+) was performed using monochromatic Al-Kα radiation (1486.6 eV) to evaluate the oxidation states of various species present within the catalysts. The calibration standard was the C 1 s peak at 284.8 eV. The infrared spectra were examined using Fourier Transform Infrared Spectroscopy (FTIR, TENSOR-37, Bruker Co., Billerica, MA, USA) operated in Attenuated Total Reflectance (ATR) mode across the wavenumber range of 4000–500 cm^−1^.

### 2.4. Transesterification Reaction

To avoid the deactivation of catalysts caused by adsorbed water, all catalysts underwent vacuum drying at 80 °C overnight prior to the transesterification reaction. The transesterification was conducted in a 250 mL three-necked reactor, featuring continuous mechanical stirring under atmospheric pressure. Initially, waste cooking oil and methanol were mixed and heated for 60 min to reach a target temperature of 65 °C. Following this, a specific amount of catalyst is added to the preheated waste cooking oil to commence the transesterification reaction at the designated temperature. Various transesterification reaction parameters—including reaction temperature from 50 to 70 °C; methanol-to-oil molar ratio from 4:1 to 20:1; catalyst dosage of 1, 2, 3, 4, and 5 g; and reaction time from 1 to 8 h—were studied to determine the optimized reaction conditions. The key reaction parameters were as follows: 20 g of waste cooking oil; a stirring speed of 360 rpm; a methanol to waste cooking oil molar ratio of 16:1; a catalyst dosage of 3 g of calcined cement; and a reaction temperature of 65 °C, unless otherwise specified. The reaction was studied by varying the stirring rate, reaction temperature, the methanol-to-WCO molar ratio, and catalyst loading to obtain the optimized reaction conditions. The conversion of waste cooking oil (WCO conversion) was measured every 1 h using gas chromatography equipped with a flame ionization detector (FID) and a capillary column (RTX-65, 30 m × 0.25 mm) (GC: 7890B, Agilent Technologies, Santa Clara, CA, USA). All transesterification reactions were performed at least three times, and the reported WCO conversion was the average value with standard deviation. After each transesterification cycle, the reaction mixture was allowed to settle for 12 h, with the bottom phase containing the catalyst and glycerol, and the upper phase consisting of biodiesel and unreacted methanol. After evaporating the methanol at 65 °C, the pure biodiesel was collected for further physical and chemical analysis. The compositions of the waste cooking oil and the resulting biodiesel were examined by using GC-MS (6890N, GC/5973 MS, Agilent Technologies).

### 2.5. Orthogonal Experiment

Orthogonal experiments were used to optimize reaction conditions and analyze interactions between operating variables for biodiesel production via transesterification. Based on preliminary experimental results and literature reports, four main factors significantly influence biodiesel production: catalyst dosage, reaction temperature, reaction time, and the methanol-to-oil molar ratio. Therefore, this experiment investigated the effects of the following four factors—catalyst dosage, reaction temperature, reaction time, and methanol-to-oil mass ratio—on the conversion rate (Table 3).

### 2.6. Catalyst Reusability

The reusability of the calcined cement catalyst was assessed by conducting the transesterification reaction over several cycles. Following each cycle, the catalyst was retrieved through filtration, rinsed with ethanol to eliminate any leftover reactants or products, and dried at 110 °C prior to its reuse in the subsequent cycle. The biodiesel yield was measured for each cycle, and the catalyst’s performance was evaluated against its initial activity.

### 2.7. Kinetic Study

In transesterification, triglycerides are converted to monoalkyl esters in the presence of methanol as the reactant. This process is performed in three consecutive steps, including (i) conversion of triglycerides (TG) to diglyceride (DG), (ii) conversion of DG to monoglyceride (MG), and finally, (iii) conversion of monoglyceride to monoalkyl ester and glycerol (GL), and the reactions can be written as Equation (1):

(1) $$TG+3\;CH_{3}OH\overset{\mathrm{catalyst}}{↔}3\;Esters+GL$$

The kinetic model of this work was built on the following assumptions: (1) The rate of the non-catalyzed reactions is negligible compared with the catalyzed reactions; (2) The methanol concentration is almost unchangeable during the reaction; and (3) The final trans-esterification reaction is performed in one step and follows pseudo-first-order kinetics.

The reaction rate can be studied using the equation below:

(2) $$-r_{a}=\frac{d\left[TG\right]}{dt}=k[TG]{\left[ROH\right]}^{3}$$

where k is the reaction constant, [TG] is the triglyceride concentration, and [ROH] is the methanol concentration. Since the methanol concentration is almost unchangeable during the reaction and k1 = k[ROH]^3^, the equation for the reaction rate can be rewritten as follows:

(3) $$-r_{a}=\frac{d\left[TG\right]}{dt}=k_{1}[TG]$$

(4) $$\ln{[TG}_{0}]-\ln[\mathrm{TG}]=k_{1}t$$

(5) $$\left[TG]={[TG}_{0}]\times (1-X\right)$$

(6) $$-\ln(1-X)=k_{1}t$$

X and [TG]_0_ refer to WCO conversion and the initial concentration of triglyceride.

The transesterification was tested at different temperatures, and the reaction rate constant k_1_ at different reaction temperatures can be obtained, and the apparent activation energy of the reaction can be calculated using the Arrhenius equation (Equation (7)):

(7) $$\ln k=-E_{a}/RT+\ln A$$

where k is the reaction rate constant (h^−1^), Ea is the apparent activation energy (kJ mol^−1^), A is the pre-exponential factor (h^−1^), R is the universal gas constant, and T is the reaction temperature (K).

### 2.8. Physicochemical Properties of Biodiesel

Upon the conclusion of the transesterification cycle, the reaction mixtures were permitted to cool to ambient temperature and subsequently allowed to settle, facilitating the separation of the liquid phase (which comprises biodiesel, methanol, and unreacted waste cooking oil) from the solid phase of calcined cement. The liquid phase was then transferred into a separating funnel and left overnight to promote the separation of methanol from the biodiesel. Following this, the raw biodiesel was purified by decompression distillation in a rotary evaporator under vacuum (10 ± 1 mm Hg) at 50 °C, with the objective of removing excess methanol and other impurities. Additional purification of the raw biodiesel was achieved through continuous washing, drying, and further decompression distillation to eliminate residual excess methanol and impurities, yielding refined biodiesel, which was subsequently tested for its physicochemical properties. The viscosity at 40 °C, density at 15 °C, flash point, pour point, cetane value, acid value, sulfur content, water content, and ash content of the refined biodiesel were assessed using the methodologies detailed in the handbook of analytical methods for biodiesel, as illustrated in Table 4.

## 3. Results

### 3.1. Catalyst Characterization

**Figure 1:**
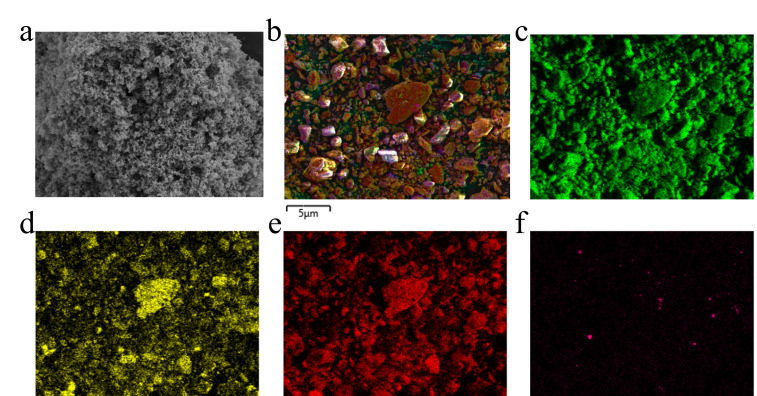
(**a**) SEM image of the calcined cement, (**b**) EDS mapping and the elements distribution of the calcined cement, (**c**) O element (green), (**d**) Ca element (yellow), (**e**) C element (red), and (**f**) Na element (pink).

The calcined cement was first characterized using scanning electron microscopy (SEM), and the results are shown in Figure 1. SEM images revealed a porous structure with a relatively uniform surface area, which is favorable for the adsorption and activation of the reactants during the transesterification process. The SEM-EDS mapping results in Figure 1b–f indicate that a homogeneous distribution of Ca, C, and O elements, with a trace amount of Na element.

**Figure 2:**
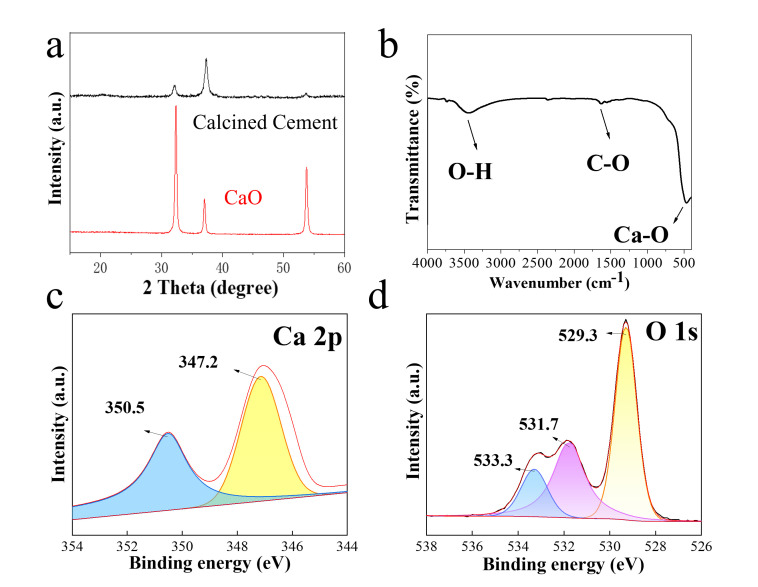
XRD (**a**), FTIR (**b**), Ca2p XPS (**c**), and O1s XPS (**d**) of the calcined cement.

The phase identity of the calcined cement was examined through X-ray diffraction (XRD). The XRD patterns for both commercial CaO and calcined cement catalysts are illustrated in Figure 2a. The commercial CaO exhibited peaks corresponding to CaO crystalline phases at 2θ = 32.3, 37.3, and 53.8° (JCPDS file No. 37-1497). In contrast, the calcined cement displayed three similar patterns to those of commercial CaO, confirming the presence of CaO as the main active phase. All three peaks displayed apparently reduced peak area, indicating a lower degree of crystallinity after annealing. FTIR was used to study the chemical bonds and functional groups of the calcined cement (Figure 2b). The calcined cement surface showed three peaks at around 500 cm^−1^, 1450 cm^−1^, and 3500 cm^−1^, representing a characteristic peak of the CaO, the C-O stretching vibration of the carbon-oxygen bond (Alghodran et al., 2025 [26]), and the O-H peak, respectively. The peak at 1450 cm^−1^ mainly originates from the adsorption of carbon dioxide in the atmosphere: during cooling and subsequent storage, the alkaline CaO in the cement reacts chemically with carbon dioxide to form calcium carbonate.

Furthermore, XPS was used to investigate the electronic structure of the calcined cement. As shown in Figure 2c, the main peak of Ca 2p3/2 located at 347.2 eV and the main peak of Ca 2p1/2 located at 350.5 eV were clearly observed, consistent with the characteristic peak positions of Ca^2+^ (Mazaheri et al., 2018; Talebian Kiakalaieh et al., 2013 [27,28]). The absence of obvious satellite peaks indicates that Ca in the sample mainly exists as the oxide state of Ca^2+^, CaO. As shown in Figure 2d, O1s XPS displayed an obvious peak in 529.3 eV, which is the typical binding energy of lattice oxygen (O^2−^) in CaO, corresponding to the oxygen atom in the Ca-O bond. Two additional peaks were observed at 531.7 eV and 533.3 eV, attributable to oxygen species on the calcium oxide surface. Specifically, the 531.7 eV peak corresponds to adsorbed oxygen (such as O^2−^, O^−^) or hydroxyl oxygen (-OH) on the calcium oxide surface, formed by the reaction of CaO with water and oxygen when exposed to air. The peak at 533.3 eV is attributed to oxygen (CO_3_^2−^) in carbonate ions, originating from the reaction of CaO with CO_2_ in air to form calcium carbonate.

### 3.2. Catalytic Activity of the Calcined Cement

#### 3.2.1. Effect of Reaction Time and Stirring Rate on the Batch Transesterification

**Figure 3:**
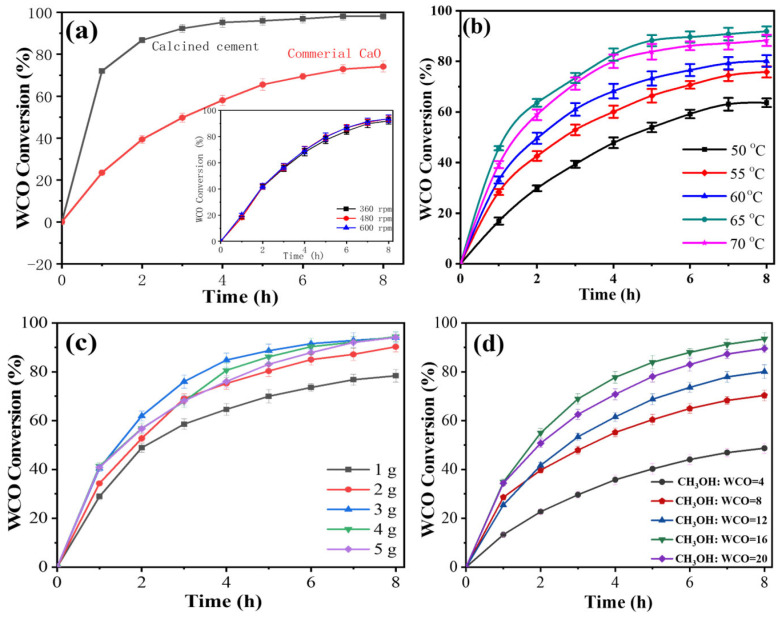
Investigation of the different parameters on the WCO conversion: reaction time and stirring speed (**a**); reaction temperature (**b**), calcined cement dosages (**c**), and methanol/WCO molar ratio (**d**).

The reaction duration plays a crucial role in determining the transesterification conversion rate, which, in turn, influences production costs. The calcined cement displayed much higher WCO conversion than commercial CaO, mainly due to its porous structure, which facilitates smoother mass transfer (Figure 3a). As illustrated in Figure 3a, with a reaction time of 1 h, the peak biodiesel production efficiency is merely 22.1%. This limitation arises from the brief reaction time, which limits catalyst particle dispersion and reduces mass transfer efficiency. Conversely, when the reaction time is extended to 8 h, the WCO conversion achieves its highest point at 91.9%. Beyond the optimal reaction duration of 8 h, any further increase in biodiesel production efficiency is negligible. The slight enhancement in biodiesel production efficiency after 8 h may be attributed to reactant clustering on the catalyst surface, which reduces the availability of catalytically active sites for transesterification. Therefore, 8 h will be used in the experiment thereafter unless otherwise stated. In addition, we studied the effect of stirring rate (360–600 rpm) on transesterification performance. We found that the stirring rate had a minimal influence on transesterification (inset of Figure 3a), suggesting that the reaction on calcined cement is kinetically, rather than diffusively, controlled.

#### 3.2.2. Effect of Reaction Temperature on the Batch Transesterification

According to the Arrhenius equation, an increase in temperature generally increases the reaction rate during transesterification. During transesterification, elevated temperatures can increase the solubility of alcohol in the oil phase, which is advantageous for the reaction by facilitating improved mixing and interaction among the various reactants. Additionally, higher temperatures may improve mass transfer, thereby promoting the diffusion of reactant molecules and enabling more efficient convergence of reactants, thereby fostering the reaction. The influence of temperature on the efficiency of biodiesel production is illustrated in Figure 3b. The findings reveal that the conversion of waste cooking oil (WCO) surged from 63% to 91% after 8 h when the reaction temperature was raised from 50 to 65 °C. However, further increasing the reaction temperature to 70 °C led to a slight reduction in WCO conversion (88.2% at 8 h), likely due to the evaporation of methanol at higher temperatures (i.e., 70 °C). Consequently, the optimal reaction temperature for this system is 65 °C, which will be used in the following experiments.

#### 3.2.3. Effect of Catalyst Dosage on the Batch Transesterification

In general, increasing the dosage of the catalyst accelerates the reaction. A higher catalyst amount offers more active sites for reactant molecules, thereby enhancing the frequency of effective collisions. As illustrated in Figure 3c, the findings demonstrate that raising the calcined cement dosage from 1 g to 3 g improved biodiesel production efficiency from 78.3% to 94.1%. However, any additional increase in the calcined cement dosage did not further enhance the biodiesel production efficiency. Considering the production cost of biodiesel, the optimal dosage of calcined cement is established to be 3 g within this reaction system.

#### 3.2.4. Effect of Methanol-to-Oil Molar Ratio on the Batch Transesterification

The molar ratio of methanol to waste cooking oil (WCO) plays a crucial role in the complete conversion of WCO into fatty acid methyl esters. Theoretically, one mole of WCO requires three moles of methanol to fully convert into three moles of fatty acid methyl esters and one mole of glycerol. However, in practical applications, an excess of methanol is typically necessary. This surplus methanol not only promotes the forward reaction and increases the reaction rate but also facilitates the phase separation of glycerol and fatty acid methyl esters (FAME). Transesterification was conducted using methanol (MeOH)/WCO molar ratios ranging from 4:1 to 20:1, as depicted in Figure 3d. The transesterification process was significantly affected by the methanol/WCO molar ratio. The conversion of WCO rose from 48% to 93.8% as the methanol/WCO molar ratio increased from 4:1 to 16:1. Nevertheless, a further increase in the methanol/WCO molar ratio led to a decline in WCO conversion, primarily because excess methanol can be adsorbed onto the surface of calcined cement, thus deactivating the catalytic materials. The optimal methanol/WCO molar ratio in a standard reaction system is 16:1.

#### 3.2.5. Reaction Kinetic Study

**Figure 4:**
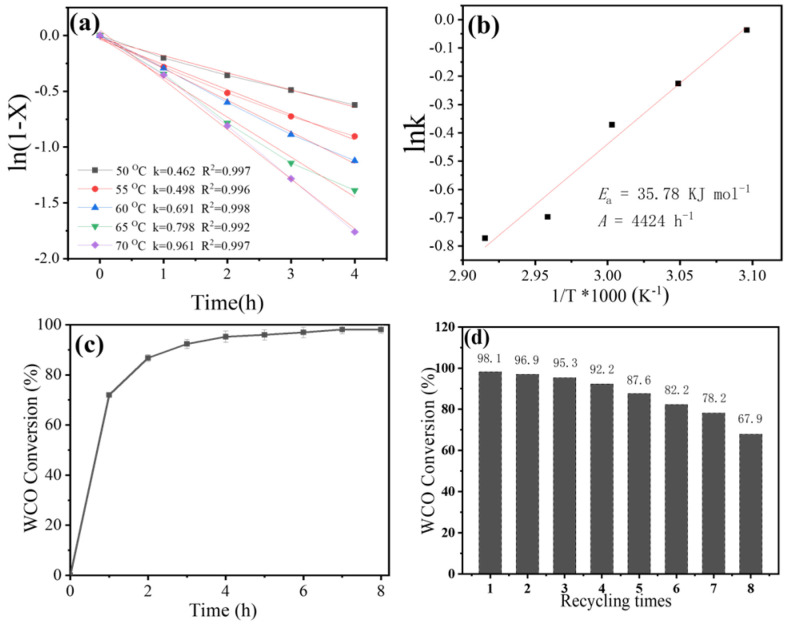
Pseudo-first-order reaction rate constants for transesterification at different temperatures (**a**), and the linear Arrhenius equation fitted between lnk and 1/T to obtain apparent activation energy Ea (**b**); Continuous transesterification results under the optimal reaction conditions (**c**); Reusability of the calcined cement under the optimal reaction conditions of WCO 20 g; reaction temperature 65 °C; methanol/WCO molar ratio 16:1; calcined cement dosage 3 g; and reaction time 8 h (**d**).

We conducted tests on the transesterification process at various temperatures and discovered that the WCO conversion during the initial 4 h aligns closely with a pseudo-first-order reaction kinetics model (R^2^ > 0.99) (Figure 4a). Consequently, the reaction rate constant k at different temperatures can be determined, and the apparent activation energy for the reaction can be calculated using the Arrhenius equation (Equation (7)). As illustrated in Figure 4b, the graph of lnk against 1/T is linear, allowing us to ascertain that Ea is 35.78 KJ mol^−1^. Based on the findings of Shi et al. and Singh [29,30], it is established that the transesterification reaction is governed by kinetic control rather than diffusion as the apparent activation energy exceeds a threshold (e.g., >20 KJ mol^−1^). The significant apparent activation energy of transesterification facilitated by calcined cement (specifically, 35.78 KJ mol^−1^) further confirms that diffusion does not play a critical role in our reaction processes.

#### 3.2.6. Orthogonal Experiment Results

To analyze the interactions among various operating variables via transesterification, an orthogonal experiment (L9 array) was conducted to examine the effects of four factors—catalyst amount, temperature, reaction time, and methanol-to-oil ratio—on waste cooking oil (WCO) conversion; the results are shown in Table 5. Among all the studied factors affecting WCO conversion, reaction time had the strongest effect, with the following order of importance: reaction time > methanol-to-oil molar ratio > catalyst dosage > reaction temperature. Among all the experiment runs, experiment No. 7 (5 g catalyst, 50 °C, 8 h, 12:1 methanol-oil molar ratio) yields the highest conversion of 86.3%.

#### 3.2.7. WCO Conversion Under the Optimal Reaction Conditions

The optimization experiments indicated that the conversion of WCO was maximized under the following reaction conditions: WCO, 20 g; reaction temperature, 65 °C; methanol/WCO molar ratio, 16:1; calcined cement dosage, 3 g; and reaction time, 8 h. These parameters led to a high biodiesel yield of approximately 98.1% (Figure 4c).

### 3.3. Catalyst Reusability

**Figure 5:**
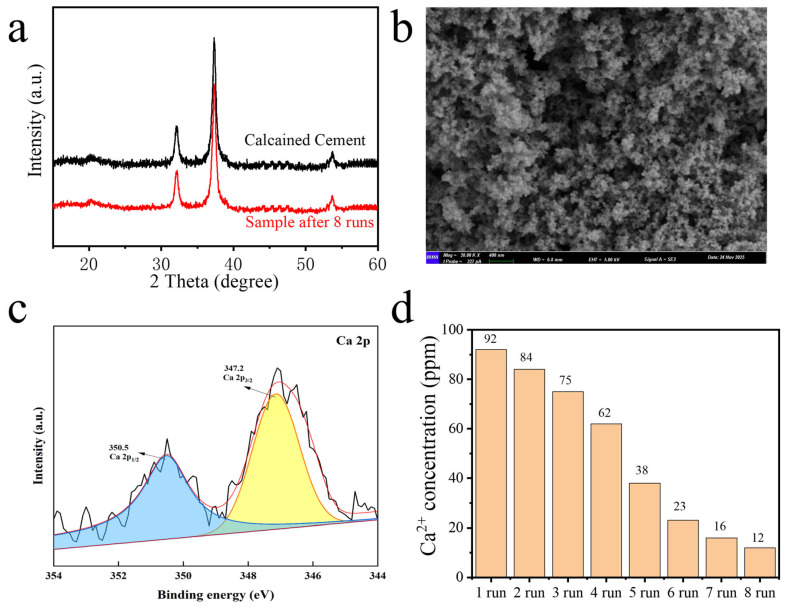
XRD of calcined cement before and after eight consecutive runs (**a**); SEM (**b**) and Ca 2p XPS (**c**) of calcined cement after eight runs; ICP-OES results of Ca^2+^ leaching in the biodiesel product phase during eight runs (**d**).

In the subsequent transesterification process, calcined cement was utilized directly without any additional treatment for eight consecutive runs, with the results illustrated in Figure 4d. The calcined cement exhibited remarkable catalytic activity for transesterification, although this activity gradually decreased after eight runs, reaching 67.9%. The deactivation of the calcined cement can be attributed to two primary factors: the adsorption of products and by-products on the catalyst’s surface, and the leaching of active catalytic sites into the solution. XRD, SEM, and XPS of calcined cement after eight runs, and ICP-OES of Ca^2+^ leaching in the product phase were studied; the results are illustrated in Figure 5. XRD, SEM, and Ca 2p XPS displayed no obvious structure changes before and after the eight runs. The ICP-OES results indicated that Ca^2+^ leaching in the biodiesel product phase at 92 ppm after the first run, decreasing gradually to 12 ppm in the 8th cycle, indicating that deactivation arises from Ca^2+^ leaching, rather than structural collapse. These results of the reusability test underscore the significant potential of calcined cement for large-scale, continuous biodiesel production.

The performance and catalytic efficiency of calcined cement have also been evaluated against other heterogeneous CaO-based catalysts employed in the production of biodiesel from different feedstocks (Table 6). The findings indicated that the heterogeneous calcined cement catalyst exhibited considerable catalytic potential and activity compared to the numerous catalysts tested. Therefore, it can be concluded that the heterogeneous waste cement is a viable option for use in the biodiesel production process derived from waste cooking oils.

### 3.4. Physicochemical Properties of the Refined Biodiesel

The physicochemical properties of the produced biodiesel are mainly determined by the compositions of fatty acid methyl esters (FAME). As shown in Table 7, the FAME compositions in the biodiesel produced mainly consisted of four components: lauric acid methyl ester, myristic acid methyl ester, palmitic acid methyl ester, and linoleic acid methyl ester. This indicates that the compositions obtained from waste cooking oil (Table 2) have been successfully converted into their corresponding esters via transesterification.

The primary fuel characteristics of the refined biodiesel were thoroughly assessed by evaluating the acid value, density, viscosity, flash point, cetane number, pour point, lower heating value, water content, and ash content. The results are detailed in Table 8 and compared with the ASTM D6751 and EN 14214 GBT 20828 standards for biodiesel. The acid value reflects the concentration of free fatty acids present in the fuel sample, which affects the density and stability of biodiesel. The acid value of the biodiesel is recorded at 0.38 mg KOH g^−1^, which complies with the maximum allowable limits across all standards. The density of biodiesel, which influences atomization efficiency, is primarily determined by the feedstock used, the ester content, and the residual methanol content. The density of the biodiesel produced at 15 °C is 878 Kg m^−3^, aligning with the biodiesel standards in both the United States and the European Union. Kinematic viscosity indicates the extent of transesterification and the purity of FAME in the produced biodiesel [41,42]. The kinematic viscosity of the biodiesel created in this study is 4.6 mm^2^ s^−1^ at 40 °C, which is within the range specified by the two standards. The flash point is related to the flammability and safety of biodiesel, and higher flash point values can mitigate the risk of fire during handling, transportation, and storage [43]. The flash point of the biodiesel produced in this study is 194 °C, which adequately meets the requirements established by the standards. The cetane number is a vital indicator of biodiesel quality, reflecting the smoothness of combustion. Consequently, an increase in cetane number is always associated with enhanced ignition quality, combustion characteristics, stability, and lower hydrocarbon emissions [44]. The cetane number of the produced biodiesel is 52 mm^2^ s^−1^, which signifies outstanding ignition quality. The pour point denotes the lowest temperature at which the fuel remains in a liquid state, thus reflecting the applicability of biodiesel in low-temperature environments [45]. Generally, the pour point values for biodiesel exceed those of mineral diesel due to the presence of various unidentified compounds formed during the transesterification process. The pour point of the biodiesel produced is 2 °C, which meets the criteria of both biodiesel standards. A high level of water and ash in biodiesel can result in significant metal corrosion, ineffective combustion, and a reduced flash point, thereby impairing its properties. The biodiesel derived from waste cooking oil contains minimal amounts of water and ash, fully meeting the minimum standards of both regulations. In conclusion, all assessed properties of the produced biodiesel conform to the standards established by the United States and the European Union, indicating that biodiesel derived from waste cooking oil can be used in diesel engines without requiring any modifications.

## 4. Discussion

In this study, the waste cement-based catalyst exhibited high activity in the transesterification reaction, possibly due to: (1) the porous structure facilitates mass transfer; and (2) the high alkalinity caused by the high CaO content on the catalyst surface. A horizontal comparison was made with typical waste-derived CaO catalysts reported over the past five years (such as eggshells, oyster shells, and steel slag) regarding raw material sources, reaction conditions, yield, and reusability. We found that waste cement achieved performance comparable to, or even better than, that of most modified CaO catalysts without the need for complex or mixed modifications, highlighting the unique advantages of waste cement as a catalyst precursor (Table 5). In addition, this study also has some limitations, such as: (i) a relatively long reaction time (8 h); (ii) a relatively high methanol/oil ratio (16:1); and (iii) the adaptability to high-acid-value waste oil has not yet been verified. These limitations point the way for further optimization. The next steps will focus on: (i) employing statistical design of experiments to model interaction effects and enable robust process scale-up; (ii) developing acid- and water-resistant bifunctional catalysts to treat low-quality oils; and (iii) conducting life cycle assessments (LCAs) to quantify environmental benefits.

## 5. Conclusions

In this work, a new heterogeneous catalyst sourced from waste cement generated by the construction industry was effectively utilized for the transesterification of waste cooking oil into biodiesel. The catalyst demonstrated notable benefits, such as low cost, environmental sustainability, and the capacity for reuse across several reaction cycles. The optimization analysis revealed that critical factors, including the methanol-to-WCO molar ratio, catalyst amount, reaction temperature, and reaction duration, were essential for enhancing biodiesel yield. The conversion of waste cooking oil (WCO) using waste cement reached 98.1% under the following optimal reaction conditions for 20 g WCO: a reaction temperature of 65 °C; a methanol/WCO molar ratio of 16:1; a calcined cement dosage of 3 g; and a reaction time of 8 h. This indicates that waste cement is a promising heterogeneous catalyst. Furthermore, the waste cement exhibited remarkable reusability even after eight consecutive catalytic cycles. The produced biodiesel exhibits high-quality, and all properties conform to the standards established by the United States and the European Union. These results imply that waste cement from construction activities is a viable catalyst for sustainable biodiesel production, providing an effective solution for transforming waste cooking oil into renewable energy.

## Figures and Tables

**Table 1:** Physicochemical properties of WCO.

Physicochemical Properties	Value
Free Fatty Acid (wt%)	1.15
Viscosity at 40 °C (mm^2^ s^−1^)	36.1
Density at 15 °C (Kg m^−3^)	924.8
Acid value (mg KOH g^−1^)	2.7
Sulfur content (ppm)	140
Water content (wt%)	0.03
Free Fatty Acid (wt%)	1.15

**Table 2:** The composition of the WCO by GC-MS.

Peak	Constituents	Free Fatty Acid *	Mole Fraction (%)
A	Lauric acid	C12: 0	12.1
B	Myristic acid	C14: 0	11.4
C	Palmitic acid	C16: 0	22.9
D	Oleic acid	C18: 1	0.7
E	Linoleic acid	C18: 2	52.9

*: the first number stands for the number of the carbon in the compound; the second number stands for the number of C=C double bonds in the compound.

**Table 3:** Experimental factors and levels.

Level	A Catalyst Dosage (g)	B Reaction Temperature (°C)	C Reaction Time (h)	D Methanol-to-Oil Molar Ratio
1	1	50	4	4:1
2	3	60	6	12:1
3	5	70	8	20:1

**Table 4:** ASTM standards for biodiesel physicochemical properties test.

Physicochemical Properties	ASTM Methods
Viscosity at 40 °C	D445
Density at 15 °C (Kg m^−3^)	D7042
Flash point (°C)	D93
Pour point (°C)	D97-05
Cetane value (mm^2^ s^−1^)	D613
Acid value (mg KOH g^−1^)	D664-07
Sulfur content (wt%)	D5453
Water content (wt%)	D1796
Ash content (wt%)	D482

**Table 5:** Results of orthogonal experiments.

NO.	Catalyst Dosage (g)	Reaction Temperature (°C)	Reaction Time (h)	Methanol-to-Oil Molar Ratio	Biodiesel Conversion (%)
1	1	1	1	1	67.2
2	1	2	2	2	78.5
3	1	3	3	3	82.1
4	2	1	2	3	75.4
5	2	2	3	1	84.7
6	2	3	1	2	70.1
7	3	1	3	2	86.3
8	3	2	1	3	62.2
9	3	3	2	1	71.2
1-level sum	75.93	76.3	66.5	74.36	
2-level sum	76.73	75.13	75.03	78.3	
3-level sum	73.23	74.46	84.36	73.23	
Maximum value	76.73	76.3	84.36	78.3	
Minimum value	73.23	75.13	66.5	73.23	Average WCO conversion = 75.3
Range	3.5	1.17	17.86	5.07	Range sum = 27.6

**Table 6:** Comparison of the ability and performance of calcined cement catalyst with other CaO-based catalysts used in the biodiesel production process from different feedstocks.

Catalyst	Feedstock	Biodiesel Yield (%)	Temp (°C)	Time (h)	Methanol: Oil (mol/mol)	Catalyst Loading	Reusability	References
Calcined cement	Waste cooking oil (WCO)	98.1	65	8	16:1	3 g	8	This work
Eggshell-derived CaO	Waste cooking oil (WCO)	96.2	60	4	12:1	3 wt%	5	[31]
CaO@TiO_2_ (eggshell-based)	Waste soybean oil	93.7	65	3	12:1	5 wt%	5	[32]
KF/CaO (from limestone)	Palm oil	96.4	60	2.5	12:1	4 wt%	4	[33]
CaO-ZnO nanocomposite	Soybean oil	95.2	65	2	15:1	3 wt%	6	[34]
Magnetic KF/CaO/Fe_3_O_4_	WCO	94.8	60	3	12:1	5 wt%	7	[35]
Dolomite-derived CaO-MgO	Rapeseed oil	92.5	65	4	18:1	6 wt%	4	[36]
CaO loaded on activated carbon	Used frying oil	91.3	65	5	15:1	4 wt%	5	[37]
Nano-CaO from waste shells	Algae oil	90.7	70	3.5	14:1	5 wt%	4	[38]
SrO-CaO-Al_2_O_3_ mixed oxide	Sunflower oil	97.0	60	2	10:1	3 wt%	5	[39]
CaO-Al_2_O_3_	WCO	95.6	65	3	12:1	4 wt%	6	[40]

**Table 7:** GC-MS analysis of the biodiesel under optimal reaction conditions.

Peak	Constituents	Free Fatty Acid *	Mole Fraction (%)
A	Lauric acid methyl ester	C12:0	13.7
B	Myristic acid methyl ester	C14:0	11.6
C	Palmitic acid methyl ester	C16:0	21.5
D	Linoleic acid methyl ester	C18:2	53.2

*: the first number stands for the number of the carbon in the compound; the second number stands for the number of C=C double bonds in the compound.

**Table 8:** Physicochemical properties of the freshly prepared biodiesel and biodiesel standard in America, the European Union, and China.

Properties	Refined Biodiesel	ASTM D6751	EN 14214
Acid value (mg KOH g^−1^)	0.38	≤0.5	≤0.5
Density at 15 °C (Kg m^−3^)	878	880	860~900
Viscosity 40 °C (mm^2^ s^−1^)	4.6	1.9~6.0	3.5~5.0
Flash point (°C)	194	≥130	≥120
Cetane value (mm^2^ s^−1^)	52	≥47	≥51
Pour point (°C)	2	−15–10	—
Sulfur content (wt%)	0.01	≤0.05	≤0.01
Acid value (mg KOH g^−1^)	0.38	≤0.5	≤0.5
Density at 15 °C (Kg m^−3^)	878	880	860~900

## Data Availability

The dataset is available upon request from the authors.

## Abbreviations

The following abbreviations are used in this manuscript:

FAME	Fatty acid methyl esters
WCO	Waste cooking oil
NaOH	Sodium hydroxide
KOH	Potassium hydroxide
CaO	Calcium oxide
FFA	Free fatty acid
GC-MS	Gas chromatography-mass spectrometry
SEM	Scanning electron microscopy
EDS	Energy dispersive spectroscopy
XRD	X-ray diffraction
XPS	X-ray photoelectron spectroscopy
FTIR	Fourier transform infrared spectroscopy
ATR	Attenuated total reflectance
TG	Triglycerides
DG	Diglyceride
MG	Monoglyceride
